# A Randomized Controlled Trial Comparing Videoconference vs. Face-to-Face Delivery of Behavior Therapy for Youths With Tourette Syndrome in the Time of COVID-19

**DOI:** 10.3389/fpsyt.2022.862422

**Published:** 2022-05-24

**Authors:** Adriana Prato, Nicoletta Maugeri, Flavia Chiarotti, Lucia Morcaldi, Carmelo M. Vicario, Rita Barone, Renata Rizzo

**Affiliations:** ^1^Child and Adolescent Neurology and Psychiatric Section, Department of Clinical and Experimental Medicine, Catania University, Catania, Italy; ^2^Department of Cognitive Sciences, Psychology, Education and Cultural Studies, University of Messina, Messina, Italy; ^3^Center for Behavioral Sciences and Mental Health, Istituto Superiore di Sanità, Rome, Italy

**Keywords:** Tourette Syndrome, behavior therapy, COVID-19, telehealth, digital health interventions

## Abstract

**Objective:**

To evaluate the clinical effectiveness of online remote behavior therapy, compared with face-to-face therapy in reducing tics and co-occurring disorders associated with the tics in a sample of youths with Tourette Syndrome.

**Design:**

A randomized controlled trial. TS patients were randomized to receive face-to-face or online remote behavior therapy.

**Participants:**

40 children aged between 9 and 16 years affected by Tourette Syndrome.

**Results:**

Online remote and face-to-face behavior therapy are equally effective in the treatment of tics and co-occurring disorders in children and adolescents affected by Tourette Syndrome. Both groups showed an improvement in the severity of tics, obsessive-compulsive symptoms, and anxiety symptoms, as assessed by neuropsychological findings. Online remote behavior therapy was more effective for reducing depressive symptoms than face-to-face behavior therapy.

**Conclusions:**

Online remote behavior therapy is a promising tool for behavioral therapies for patients with Tourette Syndrome and may represents an alternative treatment option.

## Introduction

### Background

Tourette syndrome (TS) is a neurodevelopmental condition characterized by the presence of concomitant multiple motor tics and, at least one, vocal tic, that occurs for more than 1 year, in a patient <18 years old (1); DSM-V. The prevalence was even estimated to be 0.3–1% (2, 3), TS is more common in boys than in girls with a male-to-female ratio of 3–4/1 (4). Only 10–15% of individual patients with TS have tics only (pure TS) while the remaining patient population manifests comorbid attention deficit/hyperactivity disorder (ADHD), obsessive-compulsive behaviors/obsessive-compulsive disorder (OCB/OCD), autism spectrum disorders (ASD), learning disabilities (LD), or other psychopathologies such as conduct disorder (CD), oppositional defiant disorder (ODD), anxiety disorders (AD) and depression (5, 6). Tics and co-occurring conditions are associated with functional impairment and contribute to decreases quality of life (7, 8). The etiology is complex and multifactorial. TS is polygenic, involving multiple common risk variants combined with rare, inherited or *de novo* mutations. These as well as non-genetic factors (such as perinatal events and immunological factors) are likely to contribute to the heterogeneity of the clinical phenotype, the structural and functional brain anomalies, and the neural circuitry involvement (4). Recently, the European Society for the study of Tourette Syndrome (ESSTS) wrote guidelines for the management of TS recommending psychoeducation as the initial intervention, and behavior therapy (BT) as a first-line intervention when psychoeducation alone is insufficient (9). Two approaches, habit reversal training (HRT; and its expanded version, Comprehensive Behavioral Intervention for Tics; CBIT) and exposure with response prevention (ERP), have gatered the strongest empirical support. (10–13). In situations where BT are ineffective, not available, not age-appropriate, or not the patient's or the family's preference, then pharmacological treatments should be considered.

### The Impact of the COVID-19 Epidemic on Children With Tic Disorders

The global pandemic caused by COVID-19 has created rapid changes to how people are able to carry out their normal lives, with impacts ranging from health and mortality through to those impacts brought about by social isolation rules and localized lockdowns. The social contexts for children and young people during this last year have been markedly different to what they had experienced before. Indeed, they have been subject to disrupted education at school and university, as well as hampered transition into training or the workforce for the first time (14, 15). Early results have indicated that adolescents may show an increase in symptoms of depression and anxiety, and that these are more concerned about the government restrictions designed to contain the spread of the virus, than the virus itself (16). Thus, they need our reassurance and help in these difficult times, supported by a network of informed health-care professionals. Perceived changes in tic severity during the lockdown were also recently described in school-age patients with tic disorders (17). In addition, during the global pandemic caused by COVID-19, it was reported a dramatic increase in functional tic-like behaviors in vulnerable children and adolescents after social media exposure (18, 19).

A promising development in increasing accessibility to behavioral treatments is the use of digital health interventions (DHIs) (20). Preliminary results suggest the effectiveness of DHIs for children and adolescents affected by tic disorders (21–23). In fact, telehealth will play an increasing role in the medical follow-up of patients with TS, likely beyond the end of the pandemic. However, it will be important to establish whether this type of care will be well accepted by patients and families alike (24).

## Aim of the Study

The present study aimed to evaluate the clinical effectiveness of online remote BT (or-BT), compared with face-to-face BT (ftf-BT) in reducing tics and co-occurring disorders associated with the tics in a sample of youths with TS. The study also aimed to compare the efficacy of the two treatments in improving severity of tics and other symptoms associated.

## Materials and Methods

### Study Design

This pilot study was conducted at the Child and Adolescent Neurology and Psychiatry of the Medical and Experimental Department of Catania University. A total of 40 patients with a diagnosis TS, according to the Diagnostic and Statistical Manual for Mental Disorders (DSM-V), have been enrolled. Participants were randomly assigned to the face-to-face (ftf, *n* = 20) or online remote (or, *n* = 20) BT, using a simple randomization plan based on a random number list. Prior to enrolment, all participants provided written informed consent after receiving a complete explanation of the study and the assurance that the decision to participate in the study would not interfere with their treatment in any way. All parents gave written informed consent, and the subjects assented when possible. The study was conducted in accordance with the Declaration of Helsinki and approved by the local Ethics Committee (Catania 1) of Catania University Hospital.

### Participants

Eligible participants were patients aged 9–16 years of age with a primary diagnosis of TS according to DSM-V criteria (1), recruited from September 2020 to May 2021 at the outpatient clinic of the Child and Adolescent Neuropsychiatry Unit at Catania University Hospital. The inclusion criteria were tics of moderate severity as measured by the Yale Global Tic Severity Scale (YGTSS; >13 for subjects affected by TS and >9 for those affected by CTD) (25), and an intelligence quotient (IQ) >80. Exclusion criteria were primary psychiatric disorders different from TS, intellectual disability, previous BT for tics or initiation or adjustment of any psychotropic medication for tic within the previous 2 months. Comorbid ADHD, OCD, or AD was not considered exclusion criteria unless the disorder required immediate treatment or a change in the current treatment regimen.

### Clinical Assessment

The clinical assessment of the patients was performed at two time points during the study by a pediatric neuropsychiatrist (R.R.) with solid experience in tic disorders and possible comorbidities. Participants underwent the first assessment at baseline (T0), the second after 2 months (T1). At T0, the Wechsler Intelligence Scale for Children (WISC-IV) was administered to evaluate the IQ of patients (26). At baseline point (T0), patients were also assessed according to Yale Global Tic Severity Rating Scale (YGTSS), Children's Yale-Brown Obsessive-Compulsive Scale for Children (CY-BOCS), Premonitory Urge for Tic Scale (PUTS), Multidimensional Anxiety Scale for Children (MASC), Child Depression Inventory (CDI) and the Conners' Parent Rating Scale (CPRS). Furthermore, after 2 months (T1), changes in symptoms severity were evaluated by the difference in the YGTSS, CY-BOCS, CPRS, CDI and MASC scales.

### Measures

The YGTSS is a clinician-rated scale used to assess the motor and phonic tic severity considering the number, frequency, duration, intensity, and complexity of tics. It consists of separate motor and vocal tic checklists scored from 0 to 5 on two subscales for motor and vocal tics. The subscales were combined to produce a total tic severity score (ranging from 0 to 50). Another score ranging from 0 to 50 was assigned for global impairment due to tics (25).

The PUTS measures sensory and mental phenomena associated with premonitory urges in 10 items on a four-point scale (range 10–40). The first 6 items include itchiness, energy, pressure, tense feeling, incomplete, or a not “just right” feeling before performing a tic. The additional 4 items assess whether these feelings are experienced almost all the time before a tic, if they happen with every tic, if they go away after the tic is performed, and if subjects can stop the tics for a short period of time (27). To evaluate OCD, commonly associated with TS or CTD, the CY-BOCS, a semi-structured clinician-administered interview assessing the severity of obsessions and compulsions occurring over the past week across five areas (time, interference, distressing nature, effort to resist, control over obsessions and compulsions) was also administered (28). The CPRS is a useful tool for obtaining parental reports of childhood behavior problems that contains summary scales supporting ADHD diagnosis and quantifying ADHD severity (29). Finally, all participants completed the MASC, a self-report scale that robustly represents the factor structure of anxiety in children aged 8–18 years (30) and the Child Depression Inventory: a 27-item self-report instrument that assesses depressive symptoms in 7- to 17-year-olds (31).

### Behavior Therapy

BT was conducted according to the therapist manual developed by Verdellen et al. (32). Either HRT or ERP were conducted over eight weekly sessions. Sessions were 60 min in length. In awareness training, the therapist helps the patient to recognize the premonitory urge and to generate voluntary competing responses that are incompatible with the tic (habit reversal training) and/or increase their tolerance to the premonitory urge (exposure with response prevention). A ranking of the patient's tics is constructed according to tic severity and level of impairment, and then the patient learns to perform a voluntary movement to physically prevent performance of the tic during the competing response training. Patients were required to practice at home and parents were required to monitor tics for 15 min every day.

### Materials

To perform or-BT was used Skype©, a peer-to-peer VoIP software application providing free web-based videoconferencing and utilizing security features (including standard encryption algorithms and digital user authentication certificates). Treatment was delivered from a private clinic room, using a desktop computer and a high-speed university-based internet connection. All participants used their own home computer, high speed internet connection, and a web camera to connect with the therapist.

### Statistical Analysis

Categorical variables are summarized by absolute and percent frequencies, and differences between the two treatment groups were analyzed by the Fisher's exact probability test. Quantitative variables are summarized by means, standard deviations (SD), medians and range (minimum; maximum). We assessed the distribution of quantitative variables to determine their deviation from the normal distribution within each treatment group (Shapiro–Wilk test) and the homogeneity of variance among the two treatment groups (Levene test). Since the distribution of the test scores (YGTSS, YBOCS, MASC, CDI, CONNERS) was not normal in some treatment groups at some time points, we assessed the differences between groups and time-points by non-parametric methods. Specifically, for any subject and any variable we computed the mean (T1+T2)/2 (YGTSS_m, YBOCS_m, MASC_m, CDI_m, CONNERS_m) and the variation (T1-T2) (YGTSS_d, YBOCS_d, MASC_d, CDI_d, CONNERS_d) between the values at the two time-points. We then performed the Mann-Whitney U test to assess the difference between the two treatment groups in the mean values (main effect of treatment) and in the variations (interaction treatment-by-time), and the Wilcoxon matched paired test to assess the main effect of time. In the presence of a significant interaction treatment-by-time, we repeated the Wilcoxon test separately in the two treatment groups, applying the Bonferroni's correction to account for the two comparisons. Statistical analyses were performed using STATA release 16.0 software.

## Results

### Sample Description

In this study, we enrolled a total of 40 subjects aged 9–16 years (Mean age = 13,5 ± 2,0; male (M)/female (F) = 36:4; male = 90,0%). All patients were affected by TS. The mean age of tic onset was 5,8 ± 1,2. Among the individuals diagnosed with TS, the most common comorbid psychiatric disorders were OCD (60%, *n* = 24), LD (42,5%, *n* = 17) and anxiety disorder (42,5%, *n* = 17). None of the patients had a concomitant depression, and only one patient was also affected by epilepsy. Only seven patients (17,5%) presented “pure-TS” phenotype. Seventeen (42,5%) received a pharmacological treatment (1 drug in 9, 2 drugs in 4, 3 drugs in 4) with no good response or a partial symptoms control. Participants presented a mean IQ of 103,8 (±10,6) and a mean PUTS score of 13,3 (±2,6). Demographic data and clinical features of all participants are displayed in Table 1.

**Table 1:** Participant features.

**Variable**	**Total Sample** **(*n* = 40)**	**Online remote-BT** **(*n* = 20)**	**Face-to-face-BT** **(*n* = 20)**	***p-*** **value**
Male (%)	36 (90.0%)	18 (90.0%)	18 (90.0%)	1.000
Age (mean, SD)	13.5 (SD 2.0)	13.3 (SD 2.0)	13.8 (SD 2.0)	
Age of onset	5.8 (SD 1.2)	5.8 (SD 1.0)	5.9 (SD 1.4)	0.599
Pharmacological Treatment (yes, %)	17 (42.5%)	9 (45.0%)	8 (40.0%)	1.000
**Pharmacological Treatment (** ***n*** **, %)**				0.227
0 drug	23 (57.5%)	11 (55.0%)	12 (60.0%)	
1 drug	9 (22.5%)	4 (20.0%)	5 (25.0%)	
2 drugs	4 (10.0%)	4 (20.0%)	0 (0.0%)	
3 drugs	4 (10.0%)	1 (5.0%)	3 (15.0%)	
**Pharmacological Treatment (yes, %)**				
• Atypical antipsychotics	14 (35.0%)	9 (45.0%)	5 (25.0%)	0.320
• Neuroleptic drugs	3 (7.5%)	0 (0.0%)	3 (15.0%)	0.231
• SSRI	5 (12.5%)	2 (10.0%)	3 (15.0%)	1.000
• Others	7 (17.5%)	4 (20.0%)	3 (15.0%)	1.000
**Comorbid diagnosis (yes, %)**				
• TS-only	7 (17.5%)	3 (15.0%)	4 (20.0%)	1.000
• +OCD	24 (60.0%)	12 (60.0%)	12 (60.0%)	1.000
• +LD	17 (42.5%)	8 (40.0%)	9 (45.0%)	1.000
• +Anxiety	17 (42.5%)	9 (45.0%)	8 (40.0%)	1.000
Total IQ	103.8 (SD 10.6)	104.0 (SD 9.3)	103.6 (SD 12.0)	0.653
PUTS score	13.3 (SD 2.6)	13.6 (SD 2.9)	13.1 (SD 2.3)	0.622

*SD, standard deviation. p-values refer to Fisher's exact probability test in case of categorical variables (summarized by absolute and percent frequencies), and to Mann-Whitney U test in case of quantitative variables (summarized by means and SD). All tests are two-tail*.

### Baseline Characteristics

At baseline, no statistically significant differences were observed based on neuropsychological findings in the ftf- BT group vs. the or-BT group. The mean scores for YGTSS, CY-BOCS, and MASC were slightly lower in the or-BT group vs. the ftf-BT group (YGTSS: mean 25.5, SD 10.5 vs. mean 25.8, SD 7.3, *p* = 0.773; CY-BOCS: mean 22.3, SD 12.0 vs. mean 22.7, SD 12.7, *p* = 0.644; MASC: mean 35.05, SD 16.8 vs. mean 36.15, SD 15.3, *p* = 0.663). Conversely, the mean scores for CPRS and CDI were slightly higher in the or-BT group vs. the ftf-BT group (CPRS: mean 21.15, SD 22.4 vs. mean 20.15, SD 17.2, *p* = 0.363; CDI: mean 4.45, SD 1.9 vs. mean 4.3, SD 2.6, *p* = 0.574).

### YGTSS Outcome

**Figure 1:**
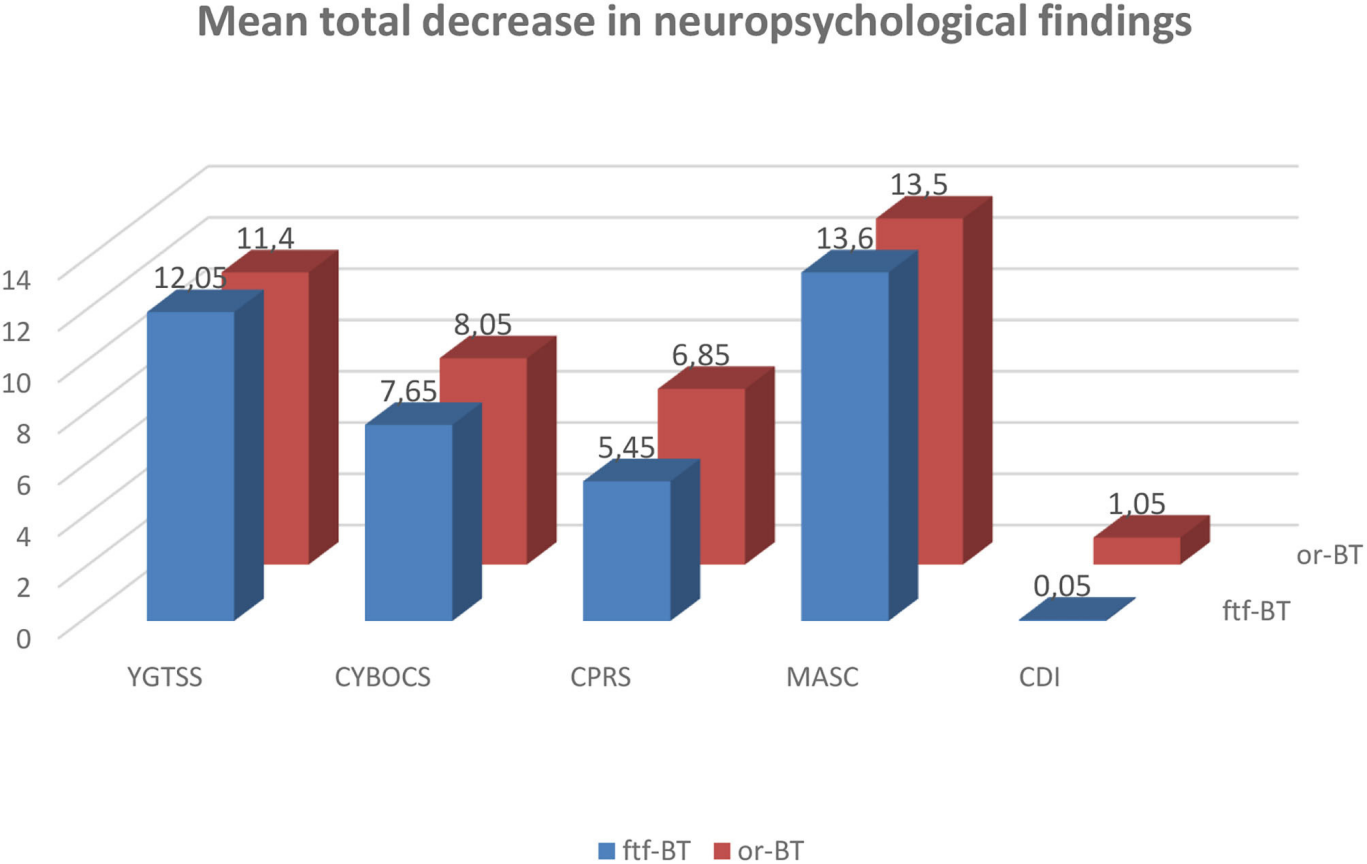
Mean total decrease in neuropsychological findings between T0 and T1.

In general, patients in both groups showed a reduction in the severity of tic symptoms, as assessed by YGTSS scores, at T1. Mean YGTSS score at 2 months after randomization was 14,1 (SD 6,3) in the or-BT -group compared with 13,7 (SD 5,35) in the ftf-BT -group. The mean total decrease in YGTSS at 2 months was 12,05 (46,8%) in the ftf-BT -group vs. 11,4 (44,7%) in the or-BT -group. No statistically significant differences were observed between the ftf-BT group vs. or-BT –group in the variation of the severity of tics as assessed by YGTSS between T0 and T1 (*p* = 0.702) (Table 2, Figure 1).

**Table 2:** Outcome of neuropsychological findings.

**Variable**	**Time**	**Online remote-BT** **(*n* = 20)**	**Face-to-face-BT** **(*n* = 20)**	***p-*** **values**	**Cohen's d**
YGTSS	T0	25.5 (SD 10.5)	25.8 (SD 7.3)	Group: 0.723	0.01
	T1	14.1 (SD 6.3)	13.7 (SD 5.4)	Time: <0.001**	0.10
	T1-T0	−11.4 (SD 6.6)	−12.1 (SD 6.0)	Group*Time: 0.702	
CYBOCS	T0T1	22.3 (SD 12.0)	22.7 (SD 12.7)	Group: 0.723	0.06
		14.3 (SD 6.6)	15.1 (SD 7.2)	Time: <0.001**	0.05
	T1-T0	−8.0 (SD 7.1)	−7.6 (SD 7.8)	Group*Time: 0.680	
CPRS	T0	21.2 (SD 22.4)	20.2 (SD 17.2)	Group: 0.260	0.02
	T1	14.3 (SD 11.7)	14.7 (SD 9.5)	Time: <0.001**	0.14
	T1-T0	−6.9 (SD 11.8)	−5.5 (SD 8.1)	Group*Time: 0.928	
CDI	T0	4.5 (SD 1.9)	4.3 (SD 2.6)	Group: 0.973	0.16
	T1	3.4 (SD 1.6)	4.3 (SD 2.5)	Time: <0.001	0.95
	T1-T0	−1.1 (SD 1.5)	−0.0 (SD 0.2)	Group*Time: 0.002** OnlineTime: 0.002** FtoF Time: 1.000	
MASC	T0	35.1 (SD 16.8)	36.2 (SD 15.3)	Group: 0.533	0.09
	T1	21.6 (SD 10.1)	22.6 (SD 5.4)	Time: <0.001**	0.01
	T1-T0	−13.5 (SD 9.1)	−13.6 (SD 12.7)	Group*Time: 0.804	

p-values refer to the non-parametric Mann-Whitney U test performed on the mean value of T0 and T1 (main effect of Group) and on the difference T1-T0 (interaction Group^*^Time), and Wilcoxon test performed in the overall group of patients (main effect of Time) or within each group (effect of Time separately assessed in the Online and in the Face-to-face groups). All tests are two-tail.

### CY-BOCS Outcome

Patients in both groups showed a reduction in the severity of obsessive-compulsive symptoms, as assessed by CYBOCS scores, at T1. Mean CYBOCS score at 2 months after randomization was 22,3 (SD 12,0) in the or-BT -group compared with 22,7 (SD 12,7) in the ftf-BT -group. The mean total decrease in CYBOCS at 2 months was 7,65 (33,7%) in the ftf-BT -group vs. 8,05 (36,1%) in the or-BT -group. No statistically significant differences were observed between the ftf-BT group vs. or-BT –group in the severity of obsessive-compulsive symptoms as assessed by CYBOCS between T0 and T1 (*p* = 0.680) (Table 2, Figure 1).

### CPRS Outcome

Patients in both groups showed a reduction in the severity of core-ADHD symptoms, as assessed by CPRS scores, at T1. Mean CPRS score at 2 months after randomization was 21,15 (SD 22,4) in the or-BT -group compared with 20,15 (SD 17,2) in the ftf-BT -group. The mean total decrease in CPRS scores at 2 months was 5,45 (27,05%) in the ftf-BT -group vs. 6,85 (32,4%) in the or-BT -group. No statistically significant differences were observed between the ftf-BT group vs. or-BT –group in the variation of the severity of these symptoms as assessed by CPRS between T0 and T1 (*p* = 0.928) (Table 2, Figure 1).

### MASC Outcome

Patients in both groups showed an improvement in MASC scores at T1. Mean MASC score at 2 months after randomization was 35,05 (SD 16,8) in the or-BT -group compared with 36,15 (SD 15,3) in the ftf-BT -group. The mean total decrease in MASC at 2 months was 13,6 (37,6%) in the ftf-BT group vs. 13,5 (38,5%) in the or-BT -group. No statistically significant differences were observed between the ftf-BT group vs. or-BT –group in the variation of the severity of anxiety symptoms as assessed by MASC between T0 and T1 (*p* = 0,804) (Table 2, Figure 1).

### CDI Outcome

Patients in both groups showed an improvement in CDI scores at T1. Mean CDI score at 2 months after randomization was 4,45 (SD 1,9) in the or-BT -group compared with 4,3 (SD 2,6) in the ftf-BT -group. The mean total decrease in CDI at 2 months was 0,05 (1,16%) in the ftf-BT group vs. 1,05 (23,6%) in the or-BT -group. Statistically significant differences were observed between the ftf-BT group vs. or-BT –group in the severity of depressive symptoms as assessed by CDI between T0 and T1 (*p* = 0.002) (Table 2, Figure 1).

## Discussion

This study investigates the efficacy of or-BT compared with ftf-BT in reducing tics and associated comorbid symptoms in youths with TS. So far, a few studies have evaluated the efficacy of BT remotely (20–22, 33). The first report about the efficacy of BT delivered via telehealth dates to a work by Himle et al. (33). These authors investigated the effectiveness of BT via videoconference in 10 TS patients compared with a face-to-face BT in 9 TS patients and demonstrated mean YGTSS reductions of 7.8 points for telehealth and 6.5 points for face-to-face (33% and 27% reductions from baseline, respectively) (33). Another 2016 RCT examined the delivery of BT via the Voice over Internet Protocol (VoIP) approach in 12 TS patient's vs. the waitlist control in 8 TS patients and found significantly greater reductions in clinician-rated and parent-reported tic severity in the VoIP-delivered BT group (21). Andrén et al. (22) also evaluated the feasibility of two existing BT protocols (HRT, ERP) into a therapist-guided and parent-guided online self-help format in a small pilot study. Both interventions resulted in reduced tic-related impairment, parent-rated tic severity and improved quality of life, and were again rated as highly acceptable, credible, and satisfactory (22). In addition, a multicentre, parallel group, single-blind RCT investigated the effectiveness of internet-delivered, therapist supported, ERP or psychoeducation and demonstrated a significant effect in treatment of tics in favor of therapist-supported ERP compared with supported psychoeducation (20). Previous studies regarding remote-BT conducted in pediatric TS patients are summarized in Table 3. Other studies have also reported the efficacy and safety of internet-delivered BT in the treatment of tics compared to ftf-BT for adults with chronic tic disorders (34, 35).

**Table 3:** Summary of studies on online-remote BT in pediatric TS patients.

**Reference**	**Design**	**Interventions**	**Patients (*n*°)**	**Mean age**	**Outcome measures**	**Results**
Himle et al. (33)	RCT	ICBT, F2F CBT	18	11.6	YGTSS	ICBT: 7.8 points reduction FCBT: 6.5 points reduction
Ricketts et al. (21)	RCT	ICBT, WL	20	12.7	YGTSS	ICBIT > WL ICBIT: 25.75 to 18.50 WL: 22.0 to 20.25
Andrén et al. (22)	RCT	BIP TIC HRT, BIP ERP	23	12.27	YGTSS	BIP TIC HRT: 23.75 to 19.00 BIP TIC ERP: 23.45 to 21.18
Hollis et al. (ORBIT) (20)	RCT	BIP TIC ERP, PE	224	12.3	YGTSS	BIP TIC ERP: 28.4 to 21.5 PE. 28.4 to 25.0

*WL, waitlist; ICBT, internet-delivered comprehensive behavioral therapy; F2F CBT, face-to face comprehensive behavioral therapy; BIP TIC HRT, internet-delivered habit reversal traning; BIP TIC ERP internet-delivered exposure and response prevention; PE, Psychoeducation; YGTSS, Yale Global Severity Scale*.

The results of this trial show that or-BT and ftf- BT are equally effective in reducing tic severity as measured by YGTSS scores. Furthermore, the mean total decrease in YGTSS at follow-up in both groups was higher (14,1 in the or-BT -group, 13,7 in the ftf-BT -group) respect to other recently reported samples (20–22, 33) (Table 3). Indeed, our results from the short follow-up assessment are more encouraging compared to the results reported in previous studies. Not only tics, but also co-occurring conditions were assessed and targeted for intervention in our study. No statistically significant differences were observed between the ftf-BT group vs. or-BT –group in the severity of obsessive-compulsive symptoms and anxiety symptoms, as assessed by neuropsychological findings. Conversely, significantly greater reductions in depressive symptoms as assessed by CDI at T1 were found in the or-BT -group relative to ftf-BT group. Participants receiving or-BT demonstrated a mean reduction in CDI score of 1,05 (23,6%), higher to that observed in the ftf-BT group (mean total decrease = - 0,05; 1,16%). Between-group differences in clinician-rated severity of depressive symptoms did reach also statistical significance (*p* = 0.010). This may be probably attributable to the major impact of lockdown on their clinical course, and to the presence of other symptoms such as sleep disturbances or somatic complaints that amplified the vulnerability due to the restrictive social isolation. It is possible to hypothesize that fear of contracting virus has amplified the vulnerability to depressed moods in these children and adolescents. Future research should examine with more details the evolution and characteristics of possible secondary symptoms during lockdown.

The current study has several limitations. First, the sample size was small, limiting statistical power and detection of within-group effect sizes. Second, our study had a short follow-up period, and so a longer interventional period than 2 months may have been required to highlight the potential benefits of or-BT compared on ftf-BT. Third, our study did not include a non-BT control group. Considering the lack of additional age-matched control-group and the relatively small sample size, the results should be considered as preliminary rather than conclusive. In addition, it would also be helpful to evaluate the effects of exposure to COVID-19-related stress on youth symptomatology. On the other hand, this study had also several strengths, including its randomized and controlled design, thoroughly considered inclusion and exclusion criteria, and the assessment of not only tics but also co-occurring conditions. In conclusion, our findings suggest that or-BT is a promising tool for behavioral therapies for patients with TS and may represents an alternative treatment option.

## Conclusions

This study suggest that or-BT is as effective as ftf-BT in the treatment of tics and co-occurring disorders in children and adolescents affected by TS or CTD. Despite this finding, further trials with larger samples are needed to confirm the beneficial effects of or-BT in treating patients with TS or CTD also affected by other comorbidities.

## Data Availability Statement

The original contributions presented in the study are included in the article/supplementary material, further inquiries can be directed to the corresponding author.

## Ethics Statement

The studies involving human participants were reviewed and approved by Local Ethics Committee (Catania 1) of Catania University Hospital. Written informed consent to participate in this study was provided by the participants' legal guardian/next of kin.

## Author Contributions

The trial was designed by RR. Treatment was provided by NM. Statistical analyses were performed by FC, in collaboration with AP. AP and LM drafted the original manuscript. RR, RB, and CV participated in constructive outline, discussions, and editing. All authors read and approved the final version of the manuscript.

## Conflict of Interest

The authors declare that the research was conducted in the absence of any commercial or financial relationships that could be construed as a potential conflict of interest.

## Publisher's Note

All claims expressed in this article are solely those of the authors and do not necessarily represent those of their affiliated organizations, or those of the publisher, the editors and the reviewers. Any product that may be evaluated in this article, or claim that may be made by its manufacturer, is not guaranteed or endorsed by the publisher.
